# Medical Society of the County of Erie and the District Attorney’s Office

**Published:** 1898-02

**Authors:** 


### BUFFALO MEDICAL JOURNAL.

A Monthly Review of Medicine and Surgery.

*EDITORS:*

THOMAS LOTHROP, M. D. - - WM. WARREN POTTER, M. D.

All communications, whether of a literary or business nature, should be addressed to the managing editor: 284 FRANKLIN STREET, BUFFALO, N. Y.

VOL. XXXVII. FEBRUARY, 1898. No. 7.

### MEDICAL SOCIETY OF THE COUNTY OF ERIE AND THE DISTRICT ATTORNEY'S OFFICE.

AT THE seventy-seventh annual meeting of the Medical Society of the County of Erie, held in Buffalo, January 11, 1898, the board of censors, through its chairman, Dr. J. B. Coakley, made an elaborate report, published in full in another column, and which merits careful reading. It is the duty of the board of censors to apprise the society and the district attorney of any violations of the medical practice law within the county of Erie, and to take preliminary steps looking toward the punishment of the offenders. In the performance of their duty during the year 1897, the censors reported several cases to the district attorney, but he did not think them sufficiently flagrant to justify prosecution, or, perhaps in some instances he may have deemed the issue not well taken. Whatever may have been his reasons, the fact remains that there were no prosecutions, hence no convictions, and the result is that Buffalo is becoming somewhat famous as a camping ground for ignorant pretenders, faith cure healers, electro-magnetic fakirs and advertising quacks.

New York has carefully undertaken the task of guarding the entrance doors to the profession of medicine by a splendid system, that has for its principal object the protection of the people against ignorance and fraud. The state says that no man shall enter upon the study of medicine until he is in possession of a preliminary education equivalent to that of a graduate of a high school, that he shall then pursue the study of medicine in a registered college during four years before he shall be entitled to a diploma. It further prescribes that the college diploma shall not be a license to practise medicine, but that its possession, obtained in the manner hereinbefore mentioned, shall be a passport to the state examination, and that having passed the latter successfully, according to the rules prescribed by the Regents of the University of the State of New York, the candidate shall be entitled to receive the state license to practise medicine and surgery, which license must be registered in the office of the clerk of the county wherein the licensee chooses to take up his residence and practise his profession. Now, if the state takes all these pains to prevent ignorant and incompetent men from entering the ranks of medicine it would be very strange if it did not intend to protect its citizens, whom it had thus carefully conducted into practice—as well as its millions of other citizens not doctors—from ignorant, incompetent and dangerous men or women, who are offering to heal the sick and maimed for a compensation, but who do not possess the legal right to do so.

Mr. Kenefick, the district attorney, for whose talent as a lawyer and whose standing as a brilliant prosecuting officer we have naught but the profoundest respect, before the ink is scarcely dry upon the censors' report makes haste, if he is correctly reported in the newspapers, to shout "malice" on the part of the censors as a defense to the course his office has pursued in regard to this subject. The suddenness with which Mr. Kenefick comes to his own defense leads us to believe, or at least to hope, that he has been misquoted. The report of the censors was a carefully prepared, painstaking and well-written document, dignified in tone and earnest in form. It deserved, to say the least, an equally careful reply in writing rather than a sudden outburst of speech delivered in the columns of the newspapers through the medium of reporters. No! Mr. Kenefick is mistaken if he thinks there is any malice in the censors' report. We do not believe there is a single member of the medical society of the county of Erie who has ought but the kindest personal feeling toward Mr. Kenefick. The attitude of the society is one of intense desire for the protection of its interests and of anxiety for the full and complete coöperation of the district attorney's office in preventing violations of the medical practice law.

The society is not satisfied to have the decision in Smith vs. Lane, based on statutes prior to 1881, held over its head like a club that in effect serves to prevent issues from being tried under statutes enacted since that date and now in force. In the county of New York there have been many arrests and a large proportion of convictions as well as a large sum collected in fines under the present law. Does it not seem strange that a statute adequate in New York is incompetent in Erie? What the medical society desires is, that a well-selected case shall be taken into the courts and fought to a finish in the hope that a judicial opinion may be obtained as to the meaning of the phrase "to practise medicine and surgery," and in the belief that a more liberal, modern and correct definition will be given than in that *bête noir* of the district attorney's office, Smith vs. Lane.

While on this subject perhaps it may not be out of place to invite Mr. Kenefick's attention to a case recently tried in Kentucky and referred to editorially in the columns of the JOURNAL, (volume XXXVII., p. 134,) issue of September, 1897. The action was brought by the Kentucky state board of health against an osteopath (whatever form of trickery that may mean) who, it was charged, had pulled and flexed the leg and thigh of a little sufferer from hip disease so that febrile action was set up that necessitated medical treatment.

Judge Thompson, before whom the case was tried, gave his opinion that this mountebank had subjected a child laboring under tuberculous disease of the hip-joint to cruel and unnecessary torture, that affected its health and necessitated the employment of a real physician to avert calamitous results. The learned judge further defined the practice of medicine as follows: "Any person who, for compensation, professes to apply any science which relates to the prevention, cure or alleviation of the diseases of the human body, is practising medicine within the meaning of the statute." This concise yet comprehensive definition, which will doubtless serve as a precedent, is broad enough to include every form of pretender from the itinerant advertising quack to the so-called christian science and faith cure healers. New York may well do homage to Kentucky, whose noble profession has driven from its borders every irregular practitioner of whatsoever kind, system or genus.

The earnestness of the Erie county society is fully attested by the fact that it proposes to employ its own attorney, whose duty it will be to prepare evidence in the cases and get it ready for the district attorney's office. This lawyer will in no wise antagonise Mr. Kenefick and his official assistants, but will be a supplementary force to their work and a coadjutor in enforcing the law. If a discreet attorney is employed by the county society, and we trust only such an one will be engaged, he has a fine opportunity to distinguish himself as an expert in medical law and to make friends with the entire profession of medicine in Erie county.

Finally, let it be understood that there can be no antagonism between the medical society of the county of Erie and the district attorney's office in this matter unless it shall be of the latter's seeking ; and that the members of the society, individually and collectively, bear the best wishes toward our brilliant district attorney, Mr. Daniel J. Kenefick.

